# Effects of Cyclooxygenase Inhibitors on Apoptotic Neuroretinal Cells

**DOI:** 10.4137/bmi.s692

**Published:** 2008-07-08

**Authors:** Anja-Kristina Brust, Holger K. Ulbrich, Gail M. Seigel, Norbert Pfeiffer, Franz H. Grus

**Affiliations:** 1 Department of Ophthalmology, Johannes Gutenberg University of Mainz, Germany; 2 Institute of Pharmacy, Department of Pharmaceutical and Medicinal Chemistry, Johannes Gutenberg University of Mainz, Germany; 3 Ross Eye Institute, Department of Ophthalmology, University of Buffalo, NY, U.S.A

**Keywords:** retinal ganglion cells, cyclooxygenase, PGE_2_, apoptosis, neuroprotection, Seldi/Maldi, biomarker neuroprotection of apoptotic neuroretinal cells

## Abstract

Glaucoma is characterized by a loss of retinal ganglion cells (RGC) which is associated with a decrease of visual function. Neuroprotective agents as a new therapeutic strategy could prevent the remaining neurons from apoptotic cell death. Previous studies have shown the involvement of the Cyclooxygenase (COX)-2 signalling in the apoptotic death of neurons. Herein we investigated the neuroprotective effect of COX-1/COX-2- and selective COX-2- inhibitors on apoptotic. R28, a neuroretinal cell line and determined the PGE_2_ levels by ELISA. Furthermore we investigated differences in protein expression in the cells after exposure to elevated pressure compared to untreated cells by ProteinChip analysis.

In addition, a protein profiling study of the cells after exposure to elevated pressure was performed. The protein expression profiles were measured by SELDI-TOF (Surface Enhanced Laser Desorption/Ionization-time of flight) Protein Chips. The protein identification was performed by mass spectrometry (MS).

It could be shown that COX-2 inhibition significantly prevented the cells from apoptosis and reduced the PGE_2_ concentrations. Selective COX-2 inhibitors were significant more potent than non-selective inhibitors or COX-1 inhibitors. We found differently expressed protein patterns in neuroretinal cells cultured at atmospheric pressure compared to those cells exposed to elevated pressure with or without celecoxib respectively. We identified three biomarkers, ubiquitin, HSP10 and NDKB, which were differently expressed in the groups. However, our data indicates a distinct neuroprotective effect of COX-2 inhibition. The local treatment with selective COX-2 inhibitors might provide an innovative strategy of therapeutic intervention for glaucoma.

## Introduction

Glaucoma is one of the most frequent causes of irreversible blindness both in industrialized countries and worldwide (Quigley, 1996; Coleman, 1999). Intraocular pressure (IOP) appears to be one of the most important risk factors for the progression of the disease. The IOP increases in persons without any subjective symptoms until irreversible damage occurs. Untreated it leads to impaired vision and ultimately to blindness in approximately 2% of the elderly population (Quigley, 1996; Coleman, 1999). Nowadays, IOP can be treated by lowering the intraocular pressure by e.g. timolol. However, raised intraocular pressure (IOP) is not the sole factor responsible for glaucomatous retinal damage. Only 10% of patients with increased IOP develop glaucomatous damage within 5 years (Kass et al. 2002), and it is commonly believed that up to 30% of patients with open angles and glaucomatous visual field defects have normal-tension glaucoma (NTG) (Hoyng et al. 2002), and there are patients where the glaucoma continues to progress despite a successful lowering of the IOP. A number of other risk factors or pathogenic mechanisms have been implicated in glaucoma, particularly in NTG, including vascular, mechanical and genetic aspects as well as myopia, endocrine abnormalities, or autoimmune phenomena (Osborne et al. 2001). These observations have been the basis for the assessment of new therapeutic options for glaucoma. Glaucomatous optic neuropathy is characterized by an apoptotic loss of retinal ganglion cells. Currently, only one multicentered and appropriately powered clinical trial, of the non-competitive *N*-methyl-D-aspartate (NMDA) antagonist, memantine (which already is Food and Drug Administration–approved for use in the United States for moderate-to-severe Alzheimer disease), assesses neuroprotection in glaucoma (Yucel et al. 2006). Memantine blocks the persistent activation of receptors by the excitatory amino acid glutamate. It has a neuroprotective effect in animal models of optic nerve injury (Wolde Mussie et al. 2002) and glaucoma (Hare et al. 2004). The hyperstimulation of NMDA receptors (Choi, 1992) lead to a massive Ca^2+^ influx that activates, among other processes, the Ca^2+^-dependent phospholipases A_2_ (PLA_2_). These PLA_2_ cleave membrane phospholipids to yield arachidonic acid, which is converted by cyclooxygenases (COXs) into prostaglandins (Hurley et al. 2002).

Two isoenzymes of COX have been identified (Vane et al. 1998). COX-1 is constitutively expressed in most tissues and is thought to mediate “housekeeping” functions. On the other hand, COX-2, an inducible enzyme, participates in the injury/inflammatory response (Vane et al. 1998; Smith et al. 1996). Among two isoenzymes of COX that have been identified, COX-2 has received more attention in the past, because it is inducible and is also constitutively expressed in brain and in a few other tissues (Vane et al. 1998; Dubois et al. 1998). Growing evidence, however, suggests that the functional significance of COX-2 is far beyond what was initially revealed (Vane et al. 1998; Dubois et al. 1998; Bazan, 2001; Ho et al. 1999). In the brain, COX-2 is expressed in discrete populations of neurons, is enriched in the cortex and hippocampus (Yamagata et al. 1993), and has been implicated in brain functions and in neurologic disorders, including stroke, seizures, and Alzheimer’s disease (Ho et al. 1999; Hewett et al. 2000; Iadecola et al. 2001; Miettinen et al. 1997; Nakayama et al. 1998).

The involvement of COX-2 in acute and chronic neurodegenerative syndromes has promoted the development of neuroprotective treatment strategies involving COX inhibitors, such as the non-steroidal anti-inflammatory drugs (NSAIDs). Although epidemiological studies suggest that NSAIDs may be protective in chronic neurodegenerative conditions (McGeer et al. 1996), however, up to now very few is known about the mechanisms of neuroprotection mediated by COX inhibitors in glaucoma.

Therefore our approach investigated the neuroprotective effects of different COX-1/COX-2- and selective COX-2- inhibitors in a cell culture model, where apoptosis was induced in a neuroretinal cell line (R28) (Table 1, Tacconelli S. et al. 2002). Furthermore, in order to assess the underlying mechanisms of possible neuroprotective effects a protein profiling study of R28 cells after exposure to elevated pressure (112 mmHg) was performed. Additionally, the PGE_2_ concentrations of the pressurized cells were analysed. The protein expression profiles were measured by SELDI-TOF (Surface Enhanced Laser Desorption/Ionization-time of flight) ProteinChips using different surfaces. The identification of the proteins which are up-or down-regulated was performed by MALDI-TOF (Matrix-Assisted Laser Desorption/Ionization-time of flight) analysis after tryptic digestion.

## Materials and Methods

### Cell cultures

R28 cells were cultured in Dulbecco’s Modified Eagle Medium (DMEM; Sigma-Aldrich Chemie GmbH, Steinheim, Germany) with 10% (v/v) FBS (fetal bovine serum, Bio Whittaker, Cambrex Bioscience, Verviers, Belgium), 1x MEM non-essential amino acids (GIBCO, Invitrogen corporation), 1x MEM vitamins (GIBCO, Invitrogen corporation), 0.37% sodium bicarbonate, 0.058% L-glutamine and 100 mg/ml gentamicin. These cells have been shown to express proteins immunoreactive with glial markers (GFAP, S-100 and vimentin), photoreceptor markers (IRBP, S-Ag, recoverin), ganglion cell marker (2G12), as well as Müller cell marker (RetG1) (Seigel et al. 1996; Seigel, 1996). Cell cultures were maintained at 37 °C and 5% CO_2_ in air in a humidified incubator. The culture medium was replaced every two days. Confluent cultures were passaged using a 1x non-enzymatic cell dissociation solution (Sigma-Aldrich, Munich, Germany).

### Reagents

Acetylic salicylic acid (ASA), meloxicam and diclofenac were purchased from Sigma-Aldrich, Munich, Germany. DFU (5,5-dimethyl-3-(3-fluorophenyl)-4-(4-methyl-sulphonyl) phenyl-2(H)-furanone), rofecoxib and celecoxib were synthesized and characterized according to literature at the Institute of Pharmacy, Department of Pharmaceutical and Medicinal Chemistry, University of Mainz, Germany (Li et al. 1999; Thérien et al. 2001; Penning et al. 1997).

### WST-1 assay

This colorimetric assay quantifies the number of metabolically active cells based on the cleavage of the tetrazolium salt WST-1(4-(3-(4-Iodophenyl)-2-(4-nitrophenyl)-2H-5-tetrazolio)-1.3-benzene disulfonate) to formazan. The assay was performed according to the manufacturer’s instructions. Absorbance from the resultant coloured solution was measured at a wavelength of 450 nm with background subtraction at 620 nm with a microplate spectrophotometer (ELISA reader, Multiskan Ascent, Thermo Electron Corporation).

### Apoptosis induction

The cells were seeded in 96 well microtiter plates (2.8 × 10^5^ cells/ml). After 24 hours, cells were grown to 40% confluency. Apoptosis was induced by serum deprivation for 48 hours. To study the neuroprotective effect of different substances the cells were incubated with serum-free medium containing the drug at different concentrations for 48 hours. After the incubation period the reduction in cell viability was measured by the colorimetric WST-1 (4-(3-(4-Iodophenyl)-2-(4-nitrophenyl)-2H-5-tetrazolio)-1.3-benzene disulfonate)-test. In another experimental setting apoptosis was induced in the cells by exposure to elevated pressure (112 mmHg) for 48 hours. Cells were placed in a closed pressurized chamber (5% CO_2_) equipped with a manometer. No significant impact on gas relationships in culture media as a result of pressurisation has been reported in similar pressure chamber based models (Agar et al. 2006). The elevated pressure was maintained for 48 hours. The cell viability was measured by the colorimetric WST-1 test. Control cells from identical passage of cell lines were simultaneously placed in an incubator at atmospheric pressure at 37 °C. The viability of the cells was examined in the presence or absence of the drugs at different concentrations dissolved in culture medium containing 10% FCS.

### Western blot analysis

Cultured cells were washed with ice-cold PBS, scraped from the culture dish in ice-cold lysis buffer containing 9.5 M urea, 2% CHAPS (3-[(3-cholamidopropyl)dimethylammonio]-1-propanesulfonate), 1% DTT (1.4 Dithiothreit) and protease inhibitors (protease inhibitor cocktail, Sigma-Aldrich Chemie GmbH, Steinheim, Germany). Lysates were sonicated and the cell homogenates were centrifuged at 13.000 × g for 10 minutes. The protein concentration in the resultant supernatants was determined by the method of Lowry and equal amounts of protein were loaded on a 12% SDS polyacrylamide gel. The proteins were transferred to nitrocellulose membranes (Protran BA 83, Schleicher and Schuell, Germany) by using a semidry blotter (Biometra). Blot efficiency was proved by reversible staining with Ponceau red to visualize proteins. The membranes were blocked with 5% dried minimal fat milk in PBS-Tween overnight. Membranes were then incubated with polyclonal antibodies against COX-1 (Alpha Diagnostic international, San Antonio, U.S.A.) and COX-2 (Calbiochem). After incubation with peroxidase-conjugated secondary antibody (Calbiochem) the signal was developed by staining with 0.05% 4-chlor-1-naphthol (Sigma-Aldrich, Munich, Germany) with 0.015% hydrogen peroxide in 20% methanol in TBS for 20 minutes. Molecular weights were estimated for each band based on the distance migrated for 10 known molecular weight standards (BenchMark, Invitrogen, Karlsruhe, Germany).

### Annexin-V propidium iodide

The cell viability was analyzed by fluorescence microscopy. We used the Annexin-V-FLUOS Staining Kit (Roche Diagnostics, Mannheim, Germany) a test kit for the detection and quantification of apoptosis and differentiation from necrosis at single cell level. The analysis is based on the staining of apoptotic and necrotic cells with Annexin-V-Fluorescein, which stains apoptotic as well as necrotic cells and propidium iodide, which stains necrotic cells only. Viable cells exclude propidium iodide and are annexin negative. The evaluation was done by fluorescence microscopy with an excitation wavelength in the range of 450–500 nm (red) and detection in the range of 515–565 nm (green). The counting of the cells was performed by using the ImageJ software (available at http://rsb.info.nih.gov/ij), by point to click counting method.

### PGE**2** quantitation assay to determine the concentration of PGE**2**

Concentrations of PGE**2** were measured using specific ELISA kits (Correlate-EIATM kit, assay designs).

The RGC were placed in PS100 cell culture dishes. At the beginning of the experiment the cells were incubated for 30 minutes with acetylsalicylic acid (ASA; 30 μM) in cell culture medium (4 ml) in order to guarantee that the PGE_2_ concentration measured was only due to the activity of the COX-2. By ASA treatment the COX-1 is acetylated irreversibly at the Serin 529 and inactivated. Subsequently, the RGC were washed 2x with 5 ml PBS and afterwards incubated with 4 ml cell culture medium alone or with 4 ml cell culture medium containing Celecoxib (5 μM) and pressurized for different periods of time (1, 3, 6, 24, and 48 h) (112 mmHg). At the end of the respective incubation period the supernatants were collected and the cells were counted after treatment with CDS solution using a Neubauer-chamber. The supernatants were centrifuged at 1200 RPM before the determination of the PGE_2_ was performed, in order to separate adherent cells.

The assay was performed according to the manufacturer’s protocol, using standard curves.

### SELDI-TOF analysis

Cell lysates were prepared in 9.5 M urea, 2% 3-[(3-cholamidopropyl)dimethylammonio]-1-propanesulfonate (CHAPS), 1% DTT (1.4 Dithiothreit) and contained protease inhibitors (protease inhibitor cocktail, Sigma-Aldrich Chemie GmbH, Steinheim, Germany). Lysis buffer was added to the plates and cells were scraped off and transferred to an eppendorf tube. After sonification and centrifugation at 13.000 × g for 10 minutes the protein concentration in the resultant supernatants was determined by the method of Lowry. Samples containing 8 μg/1μl were analyzed on a weak cation exchange surface (CM10, Ciphergen Biosystems, Fremont, CA) and a reversed-phase surface (H50, Ciphergen Biosystems, Fremont, CA). All Protein Chip Arrays were pretreated according to the standard protocols of the manufacturer. 2 μl of the samples were applicated on the spots, followed by application of 2 × 1 μl matrix (sinapic acid in 0.15% TFA (trifluoroacetic acid)/50% ACN (acetonitrile)). After drying the protein chips were measured in a SELDI-TOF (Surface Enhanced Laser Desorption/Ionization-time of flight) mass spectrometer (Ciphergen Biosystems Fremont, CA). The mass analysis was performed according to an automated data collection protocol. Spectra were calibrated with external calibrants as described previously (Grus et al. 2005). Spectral intensities were normalized by total ion current (TIC). The CE manager software was used to normalize the spectra, to automatically detect peaks, and to create the peak cluster lists. The cluster lists were exported to a statistical analysis program (Statistica). This program was used to perform a multivariate discriminant analysis to find a combination of most important biomarker (Grus et al. 2005).

### Mass spectrometry

Protein identification of differently expressed protein peaks was performed by mass spectrometry (MS). Therefore the cell lysates were separated by SDS polyacrylamide gel electrophoresis (NuPage 12% Bis-Tris-Gel; Invitrogen), stained (Colloidal Blue Staining Kit; Invitrogen) and corresponding bands were excised manually from the polyacrylamide gel and transferred into 1.5 ml reaction tubes. Excised gel pieces were treated with 150 μl of 50% methanol/10% acetic acid and agitated for 30 minutes, dehydrated with 70 μl acetonitrile for 15 minutes, followed by extraction with 50 μl of 50% formic acid/25% acetonitrile/15% isopropanol/10% water with vigorous shaking overnight. 2 μl of the extracts were applied to CM10 and H50 ProteinChip arrays and reanalyzed with the ProteinChip Reader to confirm the m/z values of the excised/extracted proteins. Afterwards, the gel extracts were vacuum dried (Concentrator; Eppendorf, Fremont, CA), 20 μl of 1% ammonia solution was added to the tubes, and the solution was vacuum dried again. 20 μl of 12 μg/1μl trypsin solution (Roche, Mannheim, Germany) were added to each tube and the samples incubated overnight at 37 °C. The peptide identification was performed using a Maldi-TOFTOF (Matrix-Assisted Laser Desorption/Ionization-time of flight) mass spectrometer (Ultraflex-Bruker Daltonics, Bremen, Germany). The Maldi spectra were exported and used for database searches with MASCOT (www.matrixscience.com/Matrix Science, Boston, MA) using the NCBI (www.ncbi.nlm.nih.gov, National Institutes of Health, Bethesda, MD) and SwissProt (http://www.wxpasy.org/Swiss Institute of Bioinformatics, Geneva, Switzerland) databases.

### Statistics

Data are represented as means of at least three identical experiments ± SEM. Statistical comparisons were performed with the unpaired Student’s Test.

## Results

### Identification of COX-1 and COX-2

**Figure 1:**
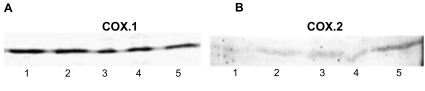
(**A**) Western blot analysis using polyclonal antibodies to COX-1 of cells treated with elevated pressure (112 mmHg). Temporal profiles of COX-1 expression are shown in the lanes: 1 = 0 h, 2 = 2 h, 3 = 6 h, 4 = 24 h, 5 = 48 h. Immunoreactivity did not change after the different time points. (**B**) Western blot analysis using polyclonal antibodies to COX-2 of cells treated with elevated pressure (112 mmHg). Temporal profiles of COX-2 expression are shown in the lanes: 1 = 0 h, 2 = 2 h, 3 = 6 h, 4 = 24 h, 5 = 48 h. Strong immunoreactivity for COX-2 was present after 48 h (lane 5); whereas COX-2 was undetectable in control cells (lane 1).

In order to verify the expression of COX-1 and COX-2 in the neuroretinal cells western blot analysis was performed. The proteins were recognized by polyclonal antibodies to COX-1 and COX-2. After treatment with elevated pressure (112 mmHg) the protein expression was measured at 0 hours, 2 hours, 6 hours, 24 hours and 48 hours. Extracts after 0 hours, 2 hours, 6 hours, 24 hours and 48 hours incubation time contained detectable amounts of COX-1 protein (approximately 70 kDa), COX-2 protein (approximately 72 kDa) was present after 48 hours of incubation whereas COX-2 was undetectable in control cells. (Fig. 1 (A, B)).

### Measurement of apoptosis inhibition by fluorescence microscopy

**Figure 2:**
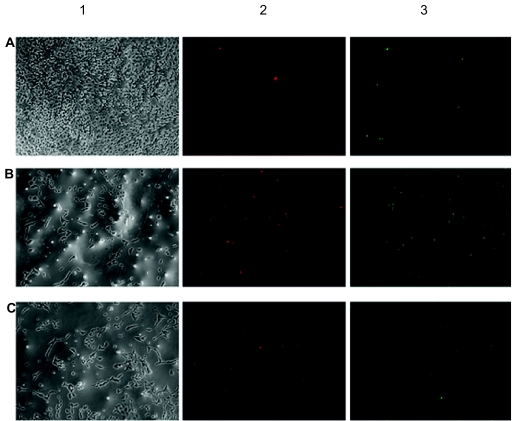
Representative phase contrast microscope images of cells that were incubated under different conditions for 48 h. (**A**) cells treated with culture medium containing 10% FCS, (**B**) cells treated with serum-free medium, (**C**) cells treated with serum-free medium containing DFU (100 μM). Column 1: phase contrast microscope images of cells under normal light conditions. Column 2: The red fluorescent images show the propidium iodide stained necrotic cells. Column 3: The green fluorescent images show the Annexin-V-stained apoptotic cells.

To be sure that the treatment with serum-free medium leads mainly to an apoptotic cell death the Annexin-V/propidium iodide staining was performed with subsequent fluorescence microscopy to differentiate between apoptotic and necrotic cells. Additionally the effect of the treatment of the cells with serum-free medium containing the specific COX-2 inhibitor DFU (100 μM) compared to serum-free medium only for 48 hours was studied (Fig. 2).

**Figure 3:**
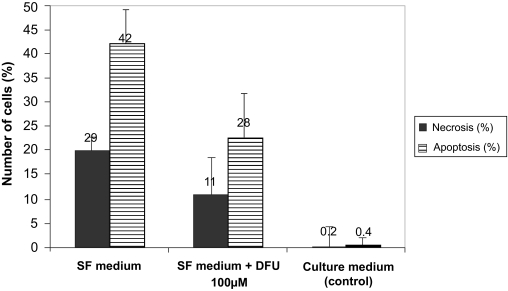
Number of apoptotic and necrotic neuroretinal cells after incubation under different conditions for 48 h. Cells treated with serum-free medium, serum-free medium containing DFU (100 μM) and culture medium containing 10% FCS. ▪ = necrotic cells in %, ⊟ = apoptotic cells in %. Total number of cells = 100% (SF medium and SF medium + DFU were significantly different from control cells treated with medium containing 10% FCS (P < 0.001 versus control)).

After the Annexin-V/propidium iodide-staining the cells were counted utilizing the ImageJ software by point to click counting method. DFU showed a distinct neuroprotective effect on apoptotic R28 cells. The incubation of the cells with serum-free medium led to 42 ± 7% apoptotic cells and 20 ± 3% necrotic cells, the incubation with serum-free medium containing 100 μM DFU caused 23 ± 7% apoptotic cells and 11 ± 13% necrotic cells, whereas control cells which were incubated with culture medium (10% FCS) showed an apoptosis rate of only 0.4 ± 2% or necrosis 0.2 ± 4% respectively (Fig. 3).

### Measurement of cell viability by the WST-1 test

**Figure 4:**
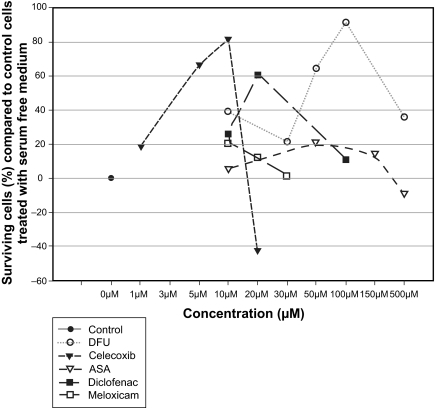
Effect of different COX-inhibitors at different concentrations dissolved in serum-free medium after 48 h treatment on apoptotic neuroretinal cells. All drugs revealed significant effects compared to control cells treated with serum-free medium only (P < 0.05 versus control). Where error bars are not shown, they were smaller than the symbol.

The neuroprotective effect of different COX-1 and/or COX-2 inhibitors was determined by the colorimetric WST-1 test. Apoptosis was induced by incubating the cells with serum-free medium for 48 hours, which is a well established method for the induction of apoptosis (Seigel et al. 2000) and led to approximately 60% reduction in cell viability. Figure 4 shows the effects of acetylic salicylic acid (ASA), meloxicam, diclofenac, DFU and celecoxib on serum deprivated cells at different concentrations.

**Figure 5:**
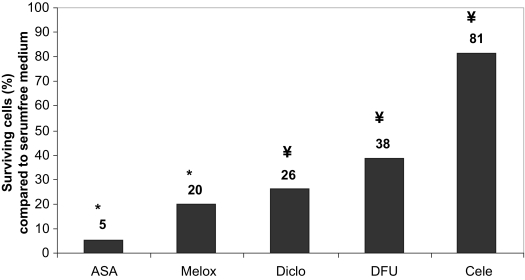
Effect of different COX-inhibitors at a concentration of 10 μM dissolved in serum-free medium after 48 h treatment on apoptotic neuroretinal cells. All drugs revealed significant effects compared to control cells treated with serum-free medium only (¥ = P < 0.001; * = P < 0.05 versus control). The neuroprotective effect of celecoxib was significantly different from all other drugs (P < 0.05 versus control). Where error bars are not shown, they were smaller than the symbol.

At the clinical achievable concentration of 10 μM (Niederberger et al. 2004) the specific COX-2 inhibitor celecoxib showed the maximal neuroprotective effect of all tested compounds, namely 81% more surviving cells compared to serum-free treated cells, followed by DFU, which protected 38% cells from apoptosis. Diclofenac could prevent 26% of the cells from undergoing apoptosis compared to incubation with serum-free medium only. The weakest effect possessed meloxicam and acetylic salicylic acid which both are unselective inhibitors of the COX isoforms. Only 20% or 5% respectively more cells survived compared to stimulation with serum-free medium (Fig. 5).

### Measurement of apoptosis after exposure to elevated pressure

**Figure 6:**
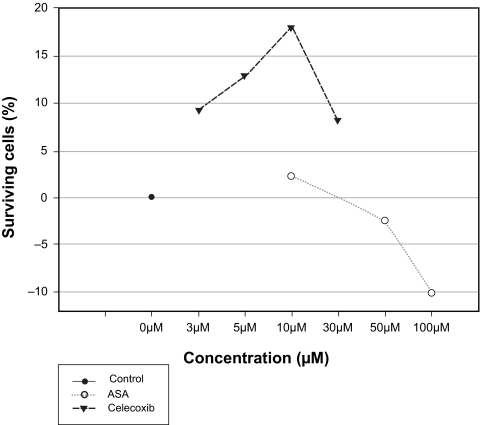
Effect of celecoxib and ASA after treatment with elevated pressure (112 mmHg) for 48 h on apoptotic neuroretinal cells. The cells were treated with celecoxib and ASA at different concentrations dissolved in culture medium containing 10% FCS. (celecoxib: P < 0.05 versus control; ASA: P > 0.05 versus control). Control cells were treated at atmospheric pressure within 48 h with culture medium containing 10% FCS only. Where error bars are not shown, they were smaller than the symbol.

The viability of the cells incubated in the presence or absence of COX-1 and/or COX-2 inhibitors at different concentrations dissolved in culture medium containing 10% FCS at elevated pressure (112 mmHg/48 h) was examined. This treatment led to apoptosis in approximately 58% cells. It was found that the selective COX-2 inhibitor celecoxib showed a significant decrease of cell death in a dose dependent manner. The specific COX-2 inhibitor celecoxib showed the maximal neuroprotective effect (18% more surviving cells vs control cells) at a concentration of 10 μM. ASA which is an unselective inhibitor of both COX isoforms could only prevent 2.3% of the cells from undergoing apoptosis (Fig. 6).

### PGE**2** quantitation

**Figure 7:**
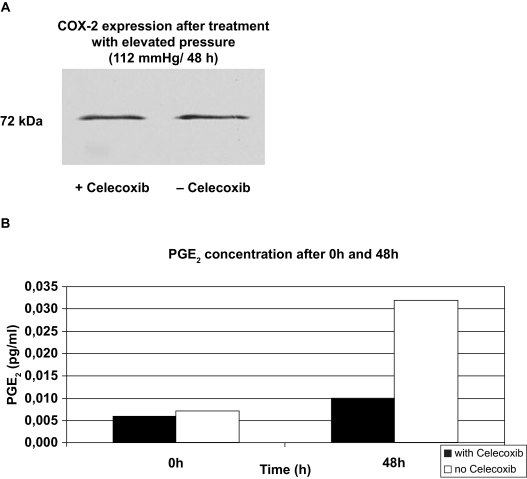
(**A**) Expression of COX-2 in pressurized RGC in the presence or absence (control cells) of celecoxib (5 μM) after 48 h incubation period. When cells expressing COX-2 were treated with celecoxib (5 μM), there was no appreciable change in the expression of COX-2. **(B)** shows the PGE_2_ synthesis of the pressurized RGC in the presence (black bars) or absence (white bars) (control cells) of celecoxib (5 μM) after 0 h or 48 h incubation period. The PGE_2_ synthesis of the celecoxib (5 μM) treated RGC was at each time point lower compared to untreated cells. RGC treated with the selective COX-2 inhibitor celecoxib (5 μM) showed a clear decrease in the PGE_2_ concentration compared to untreated cells. After 48 hour incubation period (the same incubation period which was also used for the viability tests of the cells) the celecoxib treated RGC, showed an increase of the PGE_2_ synthesis around the factor 1.7 compared to the time point 0 h. In contrast in the same period of time the PGE_2_ concentrations of the untreated RGCs increased around the factor 4.6.

In order to examine, whether the pressure treatment (112 mmHg/48 h) of the RGC leads to a COX-2 mediated increase of the prostaglandin E_2_ (PGE_2_) production, the PGE_2_ concentrations of the samples were determined after different incubation periods using the PGE_2_ kit (Correlate EIATM kit, assay Designs). Therefore the cells were prepared and incubated as described in the methods (2.7). By pre-treatment of the RGC with acetylsalicylic acid (30 μM), the COX-1 was irreversibly inhibited and it was guaranteed that the measured PGE_2_ was only due to the COX-2 activity. Figure 7A shows the expression of COX-2 in pressurized RGC in the presence or absence (control cells) of celecoxib (5 μM) after 48 h incubation period. When RGC expressing COX-2 were treated with celecoxib (5 μM), there was no appreciable change in the expression of COX-2. Figure 7B shows the PGE_2_ synthesis of the pressurized RGC in the presence (black bars) or absence (white bars) (control cells) of celecoxib (5 μM) after 0 h or 48 h incubation period. RGC treated with the selective COX-2 inhibitor celecoxib (5 μM) showed a clear decrease in the PGE_2_ concentration compared to untreated cells. After 48 hour incubation period (the same incubation period which was also used for the viability tests of the cells) the celecoxib treated RGC, showed an increase of the PGE_2_ synthesis around the factor 1.7 compared to the time point 0 h. In contrast in the same period of time the PGE_2_ concentrations of the untreated RGCs increased around the factor 4.6.

### SELDI-TOF-MS analysis

The different protein- or peptide expression in cells exposed to elevated pressure (112 mmHg) in the absence or presence of the specific COX-2 inhibitor celecoxib, respectively compared to cells cultured at atmospheric pressure for 48 h were investigated.

**Figure 8:**
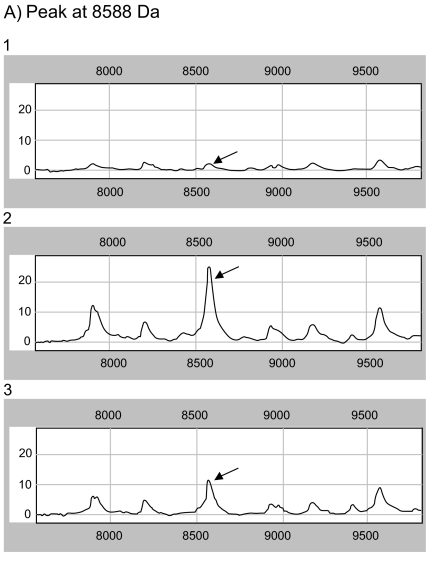

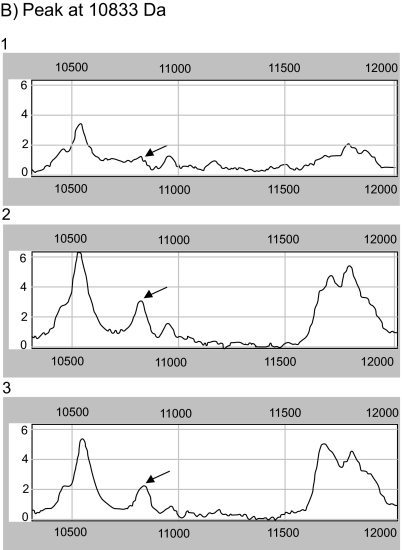

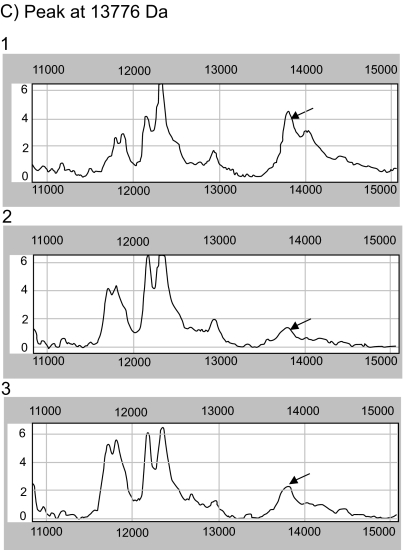
(A, B, C) SELDI-TOF patterns of cells treated at atmospheric pressure (1), cells after exposure to elevated pressure (2) and cells treated with celecoxib in a 5 μM concentration dissolved in culture medium at elevated pressure for 48 h (3), The sample containing 8 μg/1 μl were analyzed on different ProteinChip surfaces: a weak cation exchange surface (CM10) and a reversed-phase surface (H50). All Protein Chip Arrays were pretreated according to the standard protocols of the manufacturer. 2 μl of samples were applied on each ProteinChip position. After drying, 1 μl matrix solution (sinapic acid in 0.15% TFA (trifluoroacetic acid)/50% ACN (acetonitrile) ) was added twice. The ProteinChips were measured in a SELDI-TOF reader (PBSIIc, Ciphergen, Fremont, CA) with standardized laser conditions throughout the whole experiment. Spectra were normalized by total ion current (TIC). The graphs reveal the molecular weight (x-axis) vs the intensity (normalized ion current, y-axis). Each of the spectra groups above (1, 2, and 3) demonstrates the molecular weight of three different biomarkers at 8588, 10833, and 13766 Da.

The lysates of the three groups (1 = control cells at atmospheric pressure, 2 = cells at elevated pressure cultivated in culture medium without celecoxib, 3 = cells at elevated pressure cultivated in culture medium containing celecoxib (5 μM)) were used for the analysis by SELDI-TOF and MALDI-TOF (Fig. 8).

The protein profiling results were analysed by means of multivariate analysis of discriminance and artificial neural network, in order to find possible “biomarker”, which are up- or down-regulated due to the treatment options. A panel of 42 protein peaks could be demonstrated that were significantly differently expressed between the experimental groups.

The multivariate analysis of discriminance found significant differences in overall protein expression in each group compared to the control group. The complex protein pattern of the control cells cultivated without pressurization (group 1) was different from cells exposed to elevated pressure cultivated with (group 3) or without celecoxib (group 2). Furthermore the differences in protein expression comparing the group of pressurized cells without celecoxib (group 2) to those cultivated with celecoxib at a concentration of 5 μM (group 3) were investigated.

From these panels of more than 40 peaks, the analysis of discriminance revealed those as the most important to distinguish between the groups, which are listed in (Table 2).

**Figure 9:**
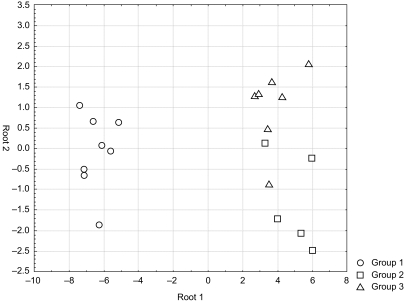
Canonical roots of the differently treated groups: 1 = cells at atmospheric pressure; 2 = cells at elevated pressure (112 mmHg); 3 = cells at elevated pressure (112 mmHg) and celecoxib (5 μM). The canonical roots were calculated in the multivariate analysis of discriminance. This graph shows the quality of separation between the groups. Each point in this plot corresponds to one single sample. The closer the points are within this graph, the more similar the protein patterns of the samples were. The graph reveals clearly a good separation between the samples treated with and without elevated pressure, but demonstrates also a significant effect between the cells treated with celecoxib or not.

The analysis of discriminance is able to calculate a parameter, the canonical roots, which can basically be used to illustrate the quality of discriminance between the groups. The closer the canonical roots of the protein pattern of each group, the more similar are the groups (Fig. 9) (Grus et al. 2006).

To identify those proteins that revealed the highest degree of difference between the groups the cell lysates were separated by SDS polyacrylamide gel electrophoresis and the corresponding bands were tryptic digested and analysed by tandem mass spectrometry. The analysis was repeated three times and yielded the same results.

The biomarker at 8588 Da was identified as ubiquitin. Ubiquitin was significantly lower expressed in those cells treated at atmospheric pressure (group 1) compared to the expression in both other groups (group 2 + 3). The expression of ubiquitin in pressurized cells without celecoxib treatment (group 2) was higher than in celecoxib treated cells (group 3) (Table 2, Fig. 8A).

**Figure 10:**
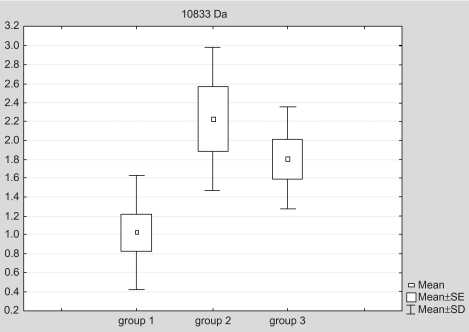
Expression of the biomarker at 10833 (mean ± SE) is plotted for the different groups: group 1 = control cells at atmospheric pressure; group 2 = cells at elevated pressure cultivated in culture medium without celecoxib; group 3 = cells at elevated pressure cultivated in culture medium containing celecoxib (5 μM).

The biomarker at 10833 Da was identified as HSP10. Its highest expression was found in cells cultured at elevated pressure without celecoxib treatment (group 2). The expression was significantly higher than in celecoxib treated cells (group 3). The lowest level was found in control cells cultivated at atmospheric pressure (group 1) (Table 2, Fig. 10 and Fig. 8B).

The biomarker at 13776 Da was identified as nucleoside diphosphate kinase B (NDKB), a protein which is decreased in those cells treated with elevated pressure (group 2 and 3) compared to the expression in control cells cultured at atmospheric pressure (group 1). The lowest protein level was found in cells treated with elevated pressure without celecoxib treatment (group 2) (Table 2, Fig. 8C).

## Discussion

Nowadays, the treatment of glaucoma is mainly based on the reduction of the intraocular pressure. Neuroprotective drugs as a new approach however could protect the neurons which still not have been damaged and stop the progression of the disease.

In our studies we could demonstrate a distinct neuroprotective effect of COX-2 inhibition on apoptotic R28, a neuroretinal cell line. We showed that the specific COX-2 inhibitors celecoxib and DFU protected serum deprivated the cells in a dose dependent manner and were significantly more potent than substances which inhibit preferentially the COX-1 or both COX-isoenzymes. Moreover, we could demonstrate a significant dose-dependent decrease of cell death of pressurized cells treated with the selective COX-2 inhibitor celecoxib. The pressure conditions of 112 mmHg were selected analogous to levels seen in acute glaucoma. It is known that elevated pressure has an impact on different aspects of cellular anatomy and physiology. Different authors demonstrated morphological changes in cell shape, alignment and processes and cytoskeletal actin redistribution in various human ocular cells (Wax et al. 2000; Kosnosky et al. 1995). However, very little is known about neuronal apoptosis and hydrostatic pressure as an isolated and independent stimulus. But different authors have already demonstrated apoptosis directly induced by hydrostatic pressure.

Previous studies have already shown the involvement of the COX-2 signaling pathways in the apoptotic death of neurons (Bagetta et al. 1998) Furthermore, an increased expression of COX-2 in retinal neurons in response to glutamate excitotoxicity, one of the mechanisms which is discussed being responsible for retinal ganglion cell death in glaucomatous optic neuropathy, was demonstrated (Strauss et al. 2002; Bazan et al. 1998). We confirmed the expression of COX-1 and COX-2 in the neuroretinal cells by western blot analysis. Earlier studies have already shown the existence of both isoforms in human eyes (Maihofner et al. 2001).

The mechanisms by which COX-2 is neurotoxic are poorly defined and controversial discussed but may be due to an increase in the production of prostanoids like prostaglandin E_2_ (PGE_2_).

Our studies showed that COX-2 positive pressurized RGCs synthesize PGE_2._ We observed differences between untreated and celecoxib-treated RGC. Untreated cell cultures had ~5 fold higher concentrations of PGE_2,_ and COX-2 inhibitor-treated cell cultures had minimal concentrations of PGE_2_: Celecoxib at a concentration of 5 μM effectively prevented the production and release of PGE_2._

On the one hand PGE_2_ (0.001–10 μM) is reported to protect cultured neurons from NMDA and glutamate induced death (Cazevieille et al. 1994). This is in line with our findings.

However, recently it was shown by Kawano et al. (Kawano et al. 2006) that PGE_2_ EP1 receptors are essential for the neurotoxicity mediated by COX-2-derived prostaglandin E_2_ in a mouse model in-vivo. Obviously the pro-death and pro-survival effects of PGE_2_ likely occur via the activation of different G-protein-coupled PGE_2_ receptors. Activation of the prostanoid EP2 receptor is normally associated with neuroprotection whereas in contrast the prostanoid EP1/EP3 receptor mediates COX-2 dependent neurotoxicity (Kawano et al. 2006).

In contrast, many studies have shown that celecoxib exerts anticacinogenic effects in various cancer cell lines by inducing apoptosis, blocking cell cycle progression and angiogenesis. (Grösch et al. 2006) This is excellently reviewed by Grösch et al. 2006. To clarify this discrepancy more studies are needed to investigate the differences in the regulation of neuronal cells and cancer cells.

However, the biomarker at 8588 Da was identified as ubiquitin. Ubiquitin is a protein which plays a central role in degradation of short-lived and regulatory proteins important in a variety of basic cellular processes, including regulation of the cell cycle, modulation of cell surface receptors and ion channels, and antigen processing and presentation. A wide variety of neurodegenerative disorders are associated with the accumulation of ubiquitinated proteins as a result of disruption of the ubiquitin/proteasome pathway in neuronal inclusions as well as in signs of inflammation (Lowe et al. 2001). Disruption of the ubiquitin/proteasome pathway can result from damaging events, such as oxidative stress and production of neurotoxic molecules, from mutations or from an aging-induced decrease in proteasome function. In these neurodegenerative disorders, the abnormal protein aggregates may, themselves, trigger the expression of inflammatory mediators, such as, COX-2 (Li et al. 2003). Our investigations showed that the highest expression of ubiquitin was found in pressurized cells without celecoxib treatment (group 2), whereas it was significantly lower expressed in those cells treated at atmospheric pressure (group 1). Accordingly COX-2 inhibition seems to diminish the disruption of the ubiquitin/proteasome pathway.

The biomarker at 10833 Da was identified as the chaperonin HSP10. Its highest expression was found in cells cultured at elevated pressure without celecoxib treatment (group 2), the lowest level was found in control cells cultivated at atmospheric pressure (group 1). HSP10 is a relatively small HSP that works in conjunction with a 60 kD co-chaperonin protein (HSP60). The HSP10-HSP60 complex is located in the mitochondrial matrix and folds newly translated proteins, re-fold partially denatured proteins, and facilitates degradation of improperly folded proteins. It also regulates transport of proteins across the mitochondrial membrane. The integrity of the mitochondrial membrane is very important since loss of mitochondrial membrane integrity can result in release of cytochrome c into the cytosol which can in turn lead to apoptosis via activation of the caspases (Hickey et al. 2000). In our investigations we found that celecoxib was able to diminish the increase of HSP10 release upon exposure to elevated pressure compared to non-treated cells.

Neurons possess a high number of mitochondria for the supply of the energy for synaptic transmission. Apoptosis of neurons for example retinal ganglion cells may be therefore closely related to mitochondrial dysfunction.

The biomarker at 13776 Da was identified as nucleoside diphosphate kinase B (NDKB), a protein which is decreased in those cells treated with elevated pressure (group 2 and 3) compared to the expression in control cells cultured at atmospheric pressure (group 1). The lowest protein level was found in cells treated with elevated pressure without celecoxib treatment (group 2).

Nucleoside diphosphate kinase (NDPK) is an enzyme that catalyzes the transfer of the terminal phosphate from nucleoside triphosphates to nucleoside diphosphates via formation of a high energy phosphorylated enzyme intermediate. Several reports have suggested that the expression and/or activity of NDPK could modulate neuronal cell proliferation, differentiation, and neurite outgrowth. It was recently shown that the protein expression levels of NDKB in both down-syndrome and Alzheimer disease showed a moderate decrease (Kim et al. 2002). These findings are in line with our investigations.

In conclusion, we identified ubiquitin, HSP10 and NDKB as biomarkers which are involved in the complex mechanism of neuroprotection mediated by COX-2 inhibition after pressure induced apoptosis of R28 cells. From more than 40 differently expressed proteins we identified in our pilot study three of the most important peaks to distinguish between the differently treated groups. Further identifications have to clarify which role the various biomarkers play in determining the fate of apoptotic retinal ganglion cells.

In summary, our findings provide further evidences for better understanding the pathological mechanisms due to elevated pressure in neurodegenerative diseases like glaucoma. Nevertheless, our cell culture model does not exactly reflect the situation in glaucomatous eyes because an elevated pressure of 112 mmHg corresponds to those seen in acute glaucoma only, but this pilot study can help to understand the mechanism how COX-2 inhibitors mediate their neuroprotective effect.

Besides further investigations in animal studies have to be performed and clinical studies have to clarify if the identified biomarkers will be found in glaucoma patients as well.

The specific COX-2 inhibitors that are nowadays systemically used in the therapy of pain are often correlated with some unwanted cardial side effects and therefore their use is currently controversially discussed among the experts (Grosser et al. 2006). However, the local treatment with selective COX-2 inhibitors in glaucoma could provide an innovative therapeutic intervention and could open up a complete new field of indication for existing COX-2 inhibitors avoiding these dangerous side effects.

## Figures and Tables

**Table 1:** This table summarizes the IC_50_ (COX-1/COX-2) ratios of different COX inhibitors.

Compound	IC_50_ Ratio (COX-1/COX-2)	Selectivity
Ibuprofen	0.5	**Non selective COX-Inhibitors**
Naproxen	0.5	
Salicylic acid	1	
Indomethacin	1.9	
Piroxicam	3.1	**Preferential COX-2 Inhibitors**
Meloxicam	11	
Diclofenac	19	
Celecoxib	30	**Selective COX-2 Inhibitors**
Rofecoxib	276	
DFU	660	

**Table 2:** The table shows the most important peaks to distinguish between the groups. The first six peaks represent the most significant signals of the panel of more than 40 peaks. The biomarker label (e.g. 13776-CM10) consists of the molecular weight (e.g. 13776 Da) and the different ProteinChips, where the biomarker was found (e.g. CM10).

Analysis of variance, marked effects are significant at p < 0.05000 In:Ex:				
	AV group 1	AV group 2	AV group 3	p
	
13776-CM10	3.111	1.402	1.442	30.001
8588-CM10	4.880	9.282	9.123	0.001
4219-CM10	1.196	4.139	3.656	0.001
4216-H50	1.432	4.882	4.805	0.002
5147-H50	1.372	0.275	0.530	0.004
10833-CM10	1.019	2.225	1.807	0.006
5319-H50	1.103	0.332	0.543	0.041
10551-CM10	5.989	5.376	4.709	0.064
4828-CM10	1.203	1.644	0.649	0.096
6246-H50	3.017	2.588	3.385	0.132
3456-CM10	0.238	0.336	0.001	0.143
